# Communication intervention to improve perceived threat of smoking-related COVID-19 and intentions to quit smoking during the COVID-19 pandemic in Thailand

**DOI:** 10.18332/tid/150363

**Published:** 2022-07-25

**Authors:** Chakkraphan Phetphum, Atchara Prajongjeep, Orawan Keeratisiroj, Kanyarat Thawatchaijareonying

**Affiliations:** 1 Department of Community Health, Faculty of Public Health, Naresuan University, Phitsanulok, Thailand; 2 Department of Community Public Health, Sirindhorn College of Public Health Phitsanulok, Phitsanulok, Thailand; 3 Tobacco Control Research Unit, Naresuan University, Phitsanulok, Thailand

**Keywords:** Thailand, cigarette, COVID-19, quit smoking, perceived threat

## Abstract

**INTRODUCTION:**

Smokers are more likely to be at risk of developing severe COVID-19. This study aimed to develop and evaluate the effect of a communication intervention for enhancing perceived threat of coronavirus 19 (COVID-19) infection associated with smoking and examine intentions to quit smoking among smokers during the COVID-19 pandemic in Thailand.

**METHODS:**

This study was of experimental design. The sample was 427 eligible smokers who were living in Kosumphi Nakhon district. They were either assigned to the intervention group (233) or control group (194). The intervention group received the communication intervention, developed based on the Health Belief Model (HBM), including education online, motivation via social networks, and communication through local mass media. The difference in mean scores between the two groups was examined using an independent t-test. Regression models were fitted to explore the factors associated with the improvement score of intention to quit smoking.

**RESULTS:**

The participants in the intervention group who received the communication intervention had a significantly higher mean score of perceived threats of smoking-induced COVID-19 (effect size=0.518, p<0.001) and had a significantly higher mean score of intentions to quit smoking (effect size=0.717, p<0.001) than in the control group. However, the number of e-cigarettes smoked per day between-groups was not significantly different (p=0.532). In the regression analysis, factors that significantly associated with the improvement score of intentions to quit smoking, included female gender (p=0.002), addicted to nicotine score (p<0.001), intervention group (p=0.010), and the improvement score of perceived threats (p=0.026).

**CONCLUSIONS:**

This community-based communication intervention could enhance the perceived threats of smoking-induced COVID-19 and increased the intentions to quit smoking among the smokers. However, further research to track the success rate of smoking cessation is still needed.

## INTRODUCTION

The coronavirus disease (COVID-19) pandemic since 2019 has caused nearly 200 million people to be infected and at least 4 million deaths across the world^1^. Thailand has been affected by the COVID-19 pandemic since 2020, with the highest number of infections at 23418 per day and the highest number of deaths at around 300 per day^1^. Greater risk factors for poor outcomes among patients with COVID-19 include older age, male sex, hypertension, diabetes, cardiovascular disease, and respiratory disease^2-4^. Furthermore, recent large-scale meta-analysis results demonstrated that smokers are more likely to experience serious health outcomes after contracting COVID-19, for example, both current and former smokers are more likely to be hospitalized after being infected and more likely to die from COVID-19 compared to non-smokers^5-9^. Therefore, encouraging smokers to quit smoking would be another strategy to reduce the chances of infection, severe symptoms, and mortality from COVID-19.

Evidence suggests that the COVID-19 pandemic is likely to impact smokers’ attitudes toward intentions to quit cigarettes, for instance, a survey in the United Kingdom, United States, Italy, India, and South Africa found that the vast majority of smokers reduced their consumption because of their intentions to quit^10-13^. Such evidence supports the notion that health concerns related to COVID-19 may be a suitable target for enhancing the effectiveness of communicating the dangers of smoking that should increase the likelihood of people quitting during the pandemic. This is in line with a recent study which showed that an increase in perceived susceptibility and severity towards COVID-19 induced by smoking was associated with higher intentions to quit smoking among current smokers^14^. In addition, messages linking smoking with COVID-19 may hold promise for discouraging smoking^15^. Therefore, intervening in communication to enhance perceived susceptibility and severity of smoking-related COVID-19 should be one of the considered strategies to help smokers quit smoking during the pandemic.

However, intervention in such communication may not be straightforward. This is due to the fact that this disease has changed the lifestyles of people suddenly and continuously, owing to social distancing measures, work-from-home (WFH) strategy, quarantines, and lockdowns that limit certain business operations (e.g. restaurants) and prohibit social gatherings. Although these strategies have a satisfactory effect on controlling and preventing the spread of COVID-19, such measures also limit the implementation of tobacco control interventions in the community. A reason for this is that the measures restricted public health agencies from performing proactive communication and health educational interventions with traditional strategies that had focused on gathering people and educating them, encouraging, and motivating smokers for quitting smoking individually.

Therefore, this action research aimed to develop and evaluate the impact of a communication intervention for enhancing perceived susceptibility and severity towards COVID-19 associated with smoking and examine intentions to quit smoking during the COVID-19 pandemic in Thailand. The original Health Belief Model (HBM)^16^ was applied as the basis for determining the content of the communication. The perceived susceptibility and severity were combined into a single construct (perceived threat), following the recommendations of Champion and Skinner^17^ and Skinner et al.^18^ that the higher the awareness of the threat, the greater the propensity for the person to avoid risky behaviors or drive the person to prevent and treat the disease. This model has been used effectively for studies to characterize the response to health threats such as swine flu and tuberculosis^19-21^. Furthermore, this present research allowed communities to participate in the development of a practical communication intervention based on the potential of the communities under social distancing and prohibited social gathering measures during the situation and context of the COVID-19 pandemic in Thailand.

## METHODS

### Study design

This was an experimental study with pre-test and post-test control group. The duration of this study was five months from July to November 2020. Additionally, this study was not funded by tobacco-related organizations or companies.

### Description of the study site

Kosamphi Nakhon is a small district in Kamphaeng Phet Province, which covers mostly rural and border areas. The total area of this district is 489.4 km^2^, divided into 3 sub-districts with 28227 citizens^22^. There were 81 people infected and one death from the COVID-19 virus^23^. As a precaution against COVID-19, this district has implemented policies such as social distancing, closing government offices, work-from-home (WFH), closing schools, quarantine, and a ban on social gatherings. We purposively selected this district for the study because it had an effective District Health Board (DHB) and this board had extensive experience in tobacco control, and the DHB committees were willing to participate in this study.

### Participants

The sample of this study was 630 current smokers who had not received smoking cessation aid, lived in Kosamphi Nakhon District, Kamphaeng Phet Province, and met four inclusion criteria: 1) had use of a smartphone, 2) could speak and read Thai, 3) could use LINE (a messaging application) and able to communicate via a video call, and 4) willing to participate in the program. The participants were categorized into two groups according to their residence: an intervention group of 350 smokers living in Lan Dokmai Tok sub-district, and a control group of 280 smokers living in Phet Chomphu sub-district. Randomization was not possible because of the geographical nature of the intervention (e.g. the use of local mass media).

**Figure 1:**
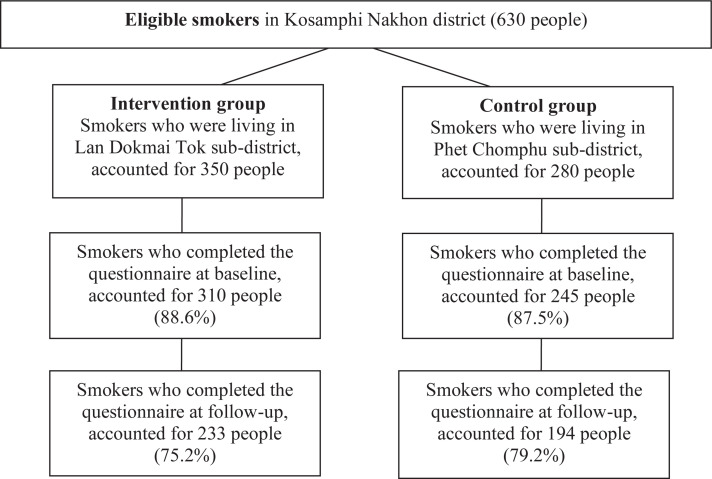
Number of samples and response rates in the intervention and control groups at baseline and follow-up

Data were collected twice from both groups of participants, for comparison between pre-study and post-study. The first dataset was collected on 26 June 2020. In this time, the researchers asked village health volunteers to send a link to an online questionnaire to the eligible smokers. A total of 310 participants of the intervention group and 245 participants of the control group completed the questionnaire, with a response rate of 88.57% and 87.5%, respectively. The second dataset of both groups was collected five months later, on 10 November 2020. The same health volunteers sent the link to the questionnaire to those who completed the questionnaire at baseline. It was found that 233 participants from the intervention group and 194 from the control group completed the questionnaire at follow-up with a response rate of 75.2% and 79.2%, respectively (Figure 1).

### Communication intervention

The communication intervention was designed and developed by the research team and co-researchers, who were the committees of the DHB of Kosamphi Nakhon district, for the intervention group. The co-researchers consisted of a district chief, heads of district government agencies, health workers, representatives of the public sectors, representatives of private sectors, and village health volunteers. A total of 40 committees of the DHB participated as co-researchers in this study. All were current non-smokers, were voluntary participants, and were not paid for their participation. This research applied Kemmis and McTaggart's cycle of action research^24^. The cycle comprised the following three phases.

*Developing a plan*

This phase aimed to allow the DHB committees to realize relevant problems, improve necessary academic competence for planning, and jointly plan to solve the determined problems. This process was conducted as a two-day workshop in July 2020. On the first day, the researchers presented information about medical findings on the relationship between smoking and infection as well as severe symptoms of COVID-19 and provided the baseline data from the survey of knowledge and perceptions of the risks of COVID-19 infection associated with smoking on 26 June 2020. On a later day, the research team and co-researchers brainstormed for proposing an alternative communication intervention that was the most appropriate for the situation and context of the COVID-19 pandemic. This step was performed by defining the content of the communication that could increase susceptibility and severity perceptions of smoking-induced COVID-19, and intentions for smoking cessation. Three communication routes were established: 1) education online, 2) motivation via social networks, and 3) communication through local mass media. The research team took responsibility for providing media and relevant equipment to support the implementation of such communication intervention.

*Implementing the plan*

This stage aimed to enable the DHB committees to bring the plan from the planning process to actual implementation in their areas. This plan could be flexible according to the situations and immediate problems and varied according to the context of the community. This process was conducted from July to October 2020 (Table 1).

**Table 1:** Timeline of milestones for intervention activities and evaluation, Thailand, 2020 (N=233)

*Activities*	*Milestones*				
	*July*	*August*	*September*	*October*	*November*
**Evaluation**	Smokers’ Survey 1 (pre-test)	-	-	-	Smokers’ Survey 2 (post-test)
**Intervention**	A				
	B				
		C	C	C	
		D	D	D	

A: Conducting a 2-day workshop to train the DHB committees and develop the plan. B: Providing education to the smokers by health workers via a video call in LINE once. C: Sending infographics to motivate and encourage smokers via LINE individually. D: Disseminating knowledge to the public through the vinyl banners installed in the community and educating through broadcast towers or community radio.

The patterns of enhancing knowledge and increasing perceived susceptibility and severity towards COVID-19 related to smoking comprised three activities as follows:

1. Education online

The health workers of Kosumpi Nakhorn District, who were responsible for a specific sub-district, directly contacted the experimental group in the communities and made an appropriate appointment for them to take part in the online education section. This section emphasized educating and questioning about the susceptibility to infection and the development of severe symptoms of the COVID-19. Additionally, all smokers were individually guided about how to stop smoking and improve their self-efficacy for successfully quitting smoking. A video call using LINE had been made to educate each smoker individually for 20 minutes.

2. Motivation through social networks

The research team and co-researchers designed and prepared infographics, using Microsoft PowerPoint, a slide show presentation program, on four topics: 1) the susceptibility to COVID-19 infection associated with smoking, 2) severe symptoms and the likelihood of death from COVID-19 associated with smoking, 3) smoking cessation guidelines, and 4) encouragement to successfully quit smoking. These infographics were then sent to the experimental group via their LINE account individually. Each infographic was sent one week apart and was resent three times. The infographic media would thus have continuously enhanced knowledge and perceptions among the participants for a total of three months.

3. Communication through local mass media

The committees of the DHB (co-researchers) took responsibility for conducting public relations and dissemination of knowledge and enhancing accurate susceptibility perceptions of becoming infected with the coronavirus associated with smoking or exposure to secondhand smoke, to the people in their responsible areas through local mass media. The media consisted of vinyl banners and broadcasting towers. The vinyl banners (80×150 cm) highlighted the main message that ‘Reduce smoking, reduce the susceptibility to COVID-19’ and were shown at the local government offices (e.g. the district office and all sub-district health promotion hospitals) as well as at public places (e.g. markets and public parks) in Lan Dokmai Tok sub-district. These banners were disseminated September to October 2020 for two months. Moreover, relevant content was produced to be used to correct knowledge and understanding about the susceptibility to coronavirus 19 infection due to smoking or exposure to secondhand smoke. This information was sent to all village headmen in the sub-district to be used as a script to be read through the broadcasting tower of every village regularly and continuously every week, twice a week.

### Reflecting and improving the plan

During the implementation of the plan, the research team and co-researchers had reflected on the results obtained from this phase in order to adjust the communication format to suit the dynamics of the COVID-19 epidemic in the community. During the communication process, the researchers found that the information that nicotine has the potential to prevent the spread of COVID-19 was shared online^25^. This message generated misperceptions among the participants and people in the communities. Therefore, the research team and DHB committees produced additional infographics to argue against such inaccurate messages and instead communicated accurate messages to these people using social media and local mass media. On the other hand, during the study, the participants of the control group who were smokers in Phet Chomphu sub-district did not receive the communication intervention to improve perceived susceptibility and severity of smoking-related COVID-19 and intentions to quit smoking during the COVID-19 pandemic.

### Measures

After receiving informed consent from all participants, data were collected using an online questionnaire generated by the research team using a Google form. The link to the questionnaire was sent to all participants via LINE and the participants then completed the questionnaire at baseline and at follow-up. The data collected were then analyzed by comparing the results before and after the implementation of the intervention, and between groups. The questionnaire consisted of the following four parts:

*Demographic characteristics*

This part included a checklist and open-ended questions assessing sex, age, education level, alcohol consumption, and type of tobacco products smoked.

*Nicotine dependence score/level*

This part contained 6 items used to access the intensity of physical addiction to nicotine using the Fagerström test for nicotine dependence^26^. All items were summed to yield a total score of 0–10 points and divided into five levels of nicotine addiction: very low (0–2), low (3–4), moderate (5), high (6–7) and very high (8–10).

*Perceived threat of smoking-related or secondhand smoke-related COVID-19*

This part included ten items (on a 5-point Likert scale) across five response options (strongly agree, somewhat agree, neither agree nor disagree, somewhat disagree, and strongly disagree). The items comprised: 1) Do you believe that smokers infected with COVID-19 are more likely to spread the disease from cigarette smoke aerosols, 2) Do you believe that smokers are more likely to get COVID-19 from the hand holding a cigarette, 3) Do you believe that smoking impairs the lungs, resulting in smokers being more vulnerable to be infected with COVID-19, 4) Do you believe that smokers infected with COVID-19 are more likely to die than non-smokers, 5) Do you believe that using the same cigarette with others increases risks of getting COVID-19 from the saliva, 6) Do you believe that exposure to secondhand smoke increases susceptibility to COVID-19, 7) Do you believe that smoking increases the ACE-2 enzyme which could allow the virus to enter the lung cells more easily, 8) Do you believe that the nicotine in cigarettes could help prevent the spread of COVID-19, 9) Do you believe that cigarette smoke aerosols could lower the lung functions, resulting in being unable to get rid of COVID-19, and 10) Do you believe that smokers could get more severe symptoms of COVID-19 than non-smokers.

*Intentions to quit smoking among smokers*

This part included a checklist question: ‘What is your intention to quit smoking score in the next 6 months?’ (5 = I have very strong intentions; 4 = I have strong intentions; 3 = I have moderate intentions; 2 = I have weak intentions, and 1 = I have no intention).

The research team assessed the quality of the questionnaire by measuring the content validity. Three qualified experts undertook this process by validating the correctness and evaluating the Index of Item-Objective Congruence (IOC). The IOC was determined with three levels of experts’ opinions on how much they were sure that a question is consistent with the specific term definitions: 1 = being sure (congruent), 0 = being unsure (questionable), and -1 = being totally unsure (incongruent). The results of the IOC analysis revealed that all questions had an IOC value between 0.67 and 1.00, which was greater than 0.5, as defined. This questionnaire was then checked for reliability by trying out on smokers in Kosamphi Nakhon District, who were not in the sample. The third part of the questionnaire with the Likert scales had a Cronbach’s alpha coefficient value of 0.793, which was greater than >0.7 (the standard) based on the newly developed measurement criteria^27^.

### Data collection

Data collection was undertaken by sending a questionnaire link to the health volunteers in the target sub-districts in Kosamphi Nakhon District, who acted as a node for distributing the link to the smokers living in their respective areas (one health volunteer was responsible for a group of 10–15 people). The first page of the online questionnaire contained information about the protection of the participants’ rights. The participants were informed that their responses to this questionnaire were anonymous, and they could refuse to participate in this research without giving any reason and not being affected. If the participants were willing to take part in this survey, they would tick a box of informed consent. After that, those who provided informed consent were allowed to complete the questionnaire four parts themselves. It took about 15–20 minutes to complete the questionnaire. All participants’ responses were then exported to an Excel file which was kept in a researcher’s coded computer. The data collected were used for the analysis of the overall results only.

### Statistical analysis

Both descriptive and inferential statistical analyses were performed using SPSS PC (Version 22.0). Most of the analyses in SPSS were descriptive, involving tables of frequencies, percentages, and appropriate summary statistics. A chi-squared test and an independent t-test were conducted to determine the degree of homogeneity of the baseline characteristics and variables between the intervention and control groups. The mean difference between the pre-test and post-test variables of perceived threats of smoking-related COVID-19, intentions to quit smoking, and number of cigarettes smoked per day between the two groups were examined using an independent t-test. The effect size was calculated as Cohen’s d.

Simple regression models were fitted to explore the association between the improvement score of intentions to quit smoking (score of post-test minus score of pre-test) and: a) characteristics of the participants including sex (female = 1, male = 0), age (years), education level (primary school or lower = 1, higher than primary school = 0), alcohol consumption (yes = 1, no = 0), type of tobacco products smoked (manufactured cigarettes = 1, hand-rolled cigarettes = 0), group (intervention group = 1, control group = 0); b) nicotine dependence score; and c) the improvement scores of perceived threats of smoking-related COVID-19 (score of post-test minus score of pre-test). A multiple regression model was fitted to explore the association between the improvement score of intentions to quit smoking and all explanatory variables that showed a statistically significant association (p<0.05) in the simple regression models. These models showed no evidence of multicollinearity, and the underlying assumptions of a normal distribution and homogeneity of variance for residuals also appeared valid. The level of statistical significance was set at 0.05.

## RESULTS

### Characteristics of participants

A total of 427 smokers: 233 from the intervention group and 194 from the control group completed the questionnaire at follow-up. The mean age of the participants was 47.5±14.0 years. Of the participants, most were male (93.9%), had secondary education (50.1%), used manufactured cigarettes (91.8%), were dependent on nicotine at a moderate level (71.0%), and 25% of the participants drank alcohol; there were no significant differences between the intervention group and the control group regarding the characteristics and nicotine dependence levels at baseline (Table 2).

**Table 2:** Homogeneity test of the characteristics and outcome variables of the intervention and control groups at baseline, Thailand, 2020 (N=427)

*Characteristics or Variables*	*Categories*	*Intervention (n=233) n (%)*	*Control (n=194) n (%)*	*p*
**Sex**	Male	220 (94.4)	181 (93.3)	0.687^a^
	Female	13 (5.6)	13 (6.7)	
**Age** (years), mean±SD		45.4±16.2	46.1±16.0	
	<60	196 (84.1)	161 (83.0)	0.794^a^
	≥60	37 (15.9)	33 (17.0)	
**Education level**	Primary school or lower	99 (42.5)	89 (45.9)	0.844^a^
	Higher than primary school	134 (57.5)	105 (54.1)	
**Drinking**	Yes	61 (26.2)	53 (27.4)	0.659^a^
	No	172 (73.8)	141 (72.6)	
**Types of smoked tobacco products**	Manufactured cigarettes	214 (91.8)	178 (91.8)	1.000^a^
	Hand-rolled cigarettes	19 (8.2)	16 (8.2)	
**Nicotine dependence level**, mean±SD		6.1±1.6	6.0±1.5	
	Very low to low	0 (0.0)	0 (0.0)	0.501^a^
	Moderate	169 (72.5)	134 (69.1)	
	High	53 (22.7)	53 (27.3)	
	Very high	11 (4.7)	7 (3.6)	
**Perceived threat of smoking-related COVID-19**, mean±SD		37.9±4.9	38.9±4.6	0.260^b^
**Intentions to quit smoking score**, median (IQR)		2.6 (0.994)	2.5 (0.861)	0.165^b^
**Cigarettes per day**, mean±SD		10.3±5.8	9.6±4.5	0.943^b^

a Chi-squared test.

b Independent t-test.

### Baseline differences between the intervention and control groups

At baseline, homogeneity tests indicated no statistical differences between the intervention and control groups in any of the following outcomes: the mean scores of perceived threats of smoking-induced COVID-19, the median scores of intentions to quit smoking, and the mean score of the number of cigarettes smoked per day (Table 2).

### The effects of the communication intervention

The changes between the pre-test and post-test outcome variables are presented in Table 3. Improvement in the mean scores of perceived threats of smoking-induced COVID-19 was significantly greater in the intervention group than in the control group (effect size=0.518; 95% CI: 1.87–3.80, p<0.001). The mean scores of intentions to quit smoking increased in the intervention group but were unchanged in the control group, and the between-group difference was significantly different (effect size=0.717; 95% CI: 0.51–0.88, p<0.001). The number of cigarettes smoked per day decreased both in the intervention group (-0.62±2.21) and the control group (-0.02±2.08), although the between-group difference was not significantly different (effect size=0.021; 95% CI: -0.40–0.59, p=0.532).

**Table 3:** Changes in the outcome variables of the two groups after the intervention, Thailand, 2020 (N=427)

*Variable*	*Intervention (n=233)*		*Control (n=194)*		*Mean difference*	*Effect size*	*95% CI*	*p*
	*Post-test Mean±SD*	*Post-test and pre-test difference*	*Post-test Mean±SD*	*Post-test and pre-test difference*				
**Perceived threat of smokingrelated COVID-19**	41.2±5.2	3.3±6.3	38.4±5.6	-0.5±6.7	2.8	0.519	1.87–3.80	<0.001*^a^
**Intentions to quit smoking score**	3.4±1.0	0.78±2.0	2.7±1.0	-0.2±1.5	0.7	0.717	0.51–0.88	<0.001*^a^
**Cigarettes per day**	9.7±4.9	-0.6±2.2	9.6±4.8	-0.1±2.1	0.1	0.021	-0.40–0.59	0.532^a^

* p<0.05

### Factors affecting the improvement score of intentions to quit smoking

Table 4 shows the results of using regression models to investigate the association between independent variables and the improvement score of intentions to quit smoking. Using simple regression models to examine each variable in turn, it was found that only sex (1 = female), nicotine dependence score, group of the participant (1 = intervention group), and the changing score of the perceived threats, were significantly associated with the improvement score of intentions to stop smoking. The multiple regression model fitted using the four independent variables was found to be significantly associated with the improvement score of intentions to quit smoking. Overall, there was a significant association with the improvement score of intentions to quit smoking (regression ANOVA, F=19.80, df=4, p<0.001, R^2^=0.16). When adjusted for the other variables in the model, the score of the intention to quit smoking was 1.09 points higher in females than males (beta=1.09; 95% CI: 0.40–1.79, p=0.002). Those who were highly addicted to nicotine had a statistically significant increase in the improvement score of intentions to quit smoking (beta=0.20, 95% CI: 0.09–0.31, p<0.001). Adjusted for other variables, the improvement score for intentions to quit smoking in the intervention group was 0.45 significantly higher than in the control group (95% CI: 0.11–0.80, p=0.010). Additionally, those who scored higher on perceived threats significantly improved their intentions to quit smoking than those with lower scores (beta=0.03, 95% CI: 0.01–0.06, p=0.026) (Table 4). Only 16% of the variation in the improvement score of intentions to quit smoking was explained by the variables in the regression model, suggesting that there may have been other unmeasured factors contributing to intentions to quit smoking.

**Table 4:** Factors affecting the improvement score of intentions to quit smoking, Thailand, 2020 (N=427)

*Variables*	*Simple regression*			*Multiple regression*		
	*Beta coefficient*	*95% CI*	*p*	*Beta coefficient*	*95% CI*	*p*
**Sex**: female^a^	0.65	0.24–1.06	0.002*	1.09	0.40–1.79	0.002*
**Age** (years)	0.00	-0.001–0.01	0.961			
**Education level:** primary school or lower^b^	-0.15	-0.35–0.05	0.138			
**Drinking:** yes^c^	-0.03	-0.25–0.20	0.801			
**Types of tobacco products:** manufactured cigarettes^d^	-0.24	-0.60–0.11	0.182			
**Nicotine dependence score**	0.08	0.02–0.15	0.014*	0.20	0.09–0.31	<0.001*
**Intervention group^e^**	0.70	0.51–0.89	<0.001*	0.45	0.11–0.80	0.010*
**Changed score of the perception threat**	0.03	0.01–0.04	<0.001*	0.03	0.01–0.06	0.026*

a Dummy variable for sex: female = 1, male = 0.

b Dummy variable for education level: primary school or lower = 1, higher than primary school = 0.

c Dummy variable for drinking: yes = 1, no = 0.

d Dummy variable for types of tobacco products: manufactured cigarettes = 1, hand-rolled cigarettes = 0.

e Dummy variable for group: intervention group = 1, control group = 0.

* p<0.05

## DISCUSSION

The community-based communication intervention improved perceived threat to smoking-related COVID-19 among the 233 smokers with a statistical significance at the 0.05 level. One explanation of this improvement could be the fact that the content provided for the smokers was up-to-date, and the issue of the relationship between smoking and COVID-19 became well known. Moreover, the performance of local government agencies as main actors in the transmission of the information may have had a positive effect on changes in such perceptions.

In addition, these issues directly affect the health of smokers and their families. Thus, they are likely to pay more attention and wish to receive more information about the issues^14^. Furthermore, this intervention focused primarily on online routes, which are practical without being contrary to social distancing measures and the ban on social gatherings. This is in line with the new lifestyles of most people since they have a smartphone with Internet access. Apart from that, some of them had to stay at home, and thus had time to access and learn from online media. Therefore, this communication intervention could enhance susceptibility perceptions among the smokers living in the communities where the COVID-19 pandemic was spreading.

It is also worth noting that this communication intervention could increase perceived threat (severe symptoms and death) of the disease, particularly in the case of the disease associated with smoking. This could be in keeping with the fact that such perceptions are consistent with their previous knowledge and experiences that lung functions of most smokers are impaired. Therefore, if smokers get infected with COVID-19, it will increase the severity of the disease. This explanation is consistent with a previous study which found that the vast majority of smokers perceived themselves to be at greater risk of severe symptoms and death due to smoking than non-smokers, while taking the risk of getting infected with COVID-19 as well^28^.

In the multiple regression analysis adjusted for participants’ characteristics, there was an increase in perceived threats associated with smoking-related COVID-19 following the communication intervention, grounded in the HBM, which was able to significantly increase the intentions to quit smoking among the participants at the 0.05 level. This result is consistent with the theoretical framework of the HBM that individuals who have improvement of perceived threat of a disease will change their risk behaviors of that disease^29-32^. This result is also in line with a previous study which suggests that smokers who have greater perceived susceptibility to infection and perceived severity of severe complications will have more desire to quit smoking. This indicates that smokers may be motivated to quit smoking due to concerns about the threats of COVID-19 that may impact them and their families in the long run as the pandemic is an ongoing crisis^14,15,^ ^29-35^.

Although this communication intervention could increase the perceived threats of the COVID-19 and increase the intentions to quit smoking among the smokers, there was no concrete positive effect on smoking reduction or quit attempt, practically. This could be due to the fact that only perceived threats are insufficient to enable individuals to change their behavior successfully. Other factors also have an impact on this change such as perceived benefits of quitting smoking with respect to COVID-19 infection and their self-efficacy in smoking cessation. Additionally, high nicotine dependence and socioeconomic status may be another reason why smokers need to rely on other aids to quit smoking successfully^36^. Therefore, an information intervention that involves an improvement in access to treatment (e.g. nicotine replacement therapy) would provide better outcomes. In addition, it may also be necessary to maintain the intensity of communication and extend the duration of the interventions, as this may increase smoking reduction or quitting attempts.

### Limitations

This research had some limitations. First, randomization was not possible because of the geographical nature of the intervention (e.g. the use of local mass media). Consequently, the intervention group and control group were not balanced. Smokers’ intentions to quit smoking might change not only because of COVID-19 communication, but also because of health warnings and education. A more balanced control group would be to have a group which received information about health risks to smokers related to other diseases (e.g. diabetes, cancer) or the risks of smoking, in general. Second, there was no monitoring and measurement of long-term outcomes of smoking cessation. The reason behind this limitation is that this research was conducted under pandemic conditions, keeping distance was thus substantial. Also, the samples had to be specific. Therefore, recruiting a large group of samples to the study and practically monitoring changes in their behaviors were restricted. Further research should consider these issues. Lastly, as this study did not collect the intervention group’s perceptions regarding public communication interventions such as radio, infographics, and vinyl banners, and also did not measure the effectiveness of different media types (e.g. social media, phone messages, community leaders), no clear conclusions were drawn. These issues thus require further study.

## CONCLUSIONS

This communication intervention could enhance perceived threat of smoking-induced COVID-19 and increase intentions to quit smoking among smokers. However, research to successfully track smoking cessation is still needed.

## Data Availability

The data supporting this research are available from the authors on reasonable request.
